# Meta-analysis of effect of vernakalant on conversion of atrial fibrillation

**DOI:** 10.1186/1756-0500-6-94

**Published:** 2013-03-13

**Authors:** He Yan, Thi Thi Aung, Zhong Guoqiang, Zhang Zhengnan, Ji Lan, Zeng Zhiyu

**Affiliations:** 1 Department of Cardiology, The First Affiliated Hospital of Guangxi Medical University, Guangxi province, China

**Keywords:** Vernakalant, Atrial fibrillation, Sinus rhythm, Conversion rate, Meta-analysis

## Abstract

**Background:**

In recent years, there has been a large amount of studies about the efficacy and safety of vernakalant or RSD1235, an antiarrhythmic agent, in treating the atrial fibrillation (AF). This study was designed to assess the efficacy and safety of vernakalant in the treatment of AF.

**Results:**

A total of 5 randomized controlled trials (RCTs) (n= 1153) met our inclusion criteria. Vernakalant was superior in achieving sinus rhythm (SR) for AF comparing to placebo or alternative anti-arrhythmic agents (relative risk [RR] = 11.56, 95% Confidence Interval [CI] = 7.12 – 18.75). There was no heterogeneity among the trials (X^2^ =0.59, P = 0.96). In analysing the adverse effects of cardiac origin, there was no significant difference between the two groups (RR= 0.90, 95% CI = 0.52 – 1.57).

**Methods:**

The Cochrane library, Pubmed NCBI, EMBASE and MEDLINE were systematically searched to identify all interventional trials of vernakalant with placebo or other antiarrhythmic drug in converting AF to SR. The primary outcome was rate of converting to SR, and the secondary outcome was the rate of adverse effects of cardiac origin due to vernakalant and the placebo or amiodarone. Meta-analyses were carried out using Mantel-Haenszel fixed-effects or random-effects models and heterogeneity was by the X^2^ test.

**Conclusion:**

In the conversion of AF to SR, vernakalant is highly effective without obviously raised side effects. Owing to only one study comparing vernakalant with amiodarone included in this study, the efficacy of vernakalant comparing to other antiarrhythmic agents needing more well-designed double-blinded RCTs to be confirmed.

## Background

Atrial fibrillation (AF) is the most common sustained arrhythmia in population [1,2], and has association with significant morbidity and mortality. AF is highly associated with 5-fold increased risk of thromboembolic strokeand 2-fold increased risk of death than the general population [3,4]. Most patients with AF do not have significant hemodynamic instability and as such, pharmacological therapy is usually the initial treatment of choice.

Currently available antiarrhythmic drugs have modest efficacy in converting atrial fibrillation (AF) to sinus rhythm (SR). Because of the pro-arrhthmic potential of presently used anti-arrhythmic agents, there is an unmet need for a more safe and effective drug that will control arrhythmias. Vernakalant is a novel antiarrhythmic drug, a sodium and ultra-rapid potassium channels blocker, prolonging atrial refractory periods and rate dependently show atrial conduction and has limited actions on ventricles [5,6]. It is available in both oral and intravenous forms. Phase III clinical trials of the intravenous formation and early Phase II studies of the oral formulation demonstrated vernakalant to be efficatious and safe in converting AF to SR. In patients with recent onset AF, vernakalant has been shown to be effective in rapid achieving and maintaining SR (51.2% to 51.7%) [7,8] in some studies. In the FDA briefing documents, it was stated that vernakalant could also provide rapid recovery of atrial fibrillation-related symptoms as such palpitations, breathlessness, chest tightness/pain, dizziness and fatigue. Vernakalant was well tolerated in atrial fibrillation patients and the treatment-related adverse effects (AEs) most commonly occurred within the first 2 hours in patients receiving vernakalant while the most occurring ones were dysgeusia, sneezing and paraesthesia.

We pooled the data extracted from the included studies that compared vernakalant with placebo or amiodarone in conversion of AF to SR to gain sufficient power to evaluate the potential differences in efficacy and safety.

## Method

### Literature search

We searched Pubmed NCBI, EMBASE, MEDLINE and the Cochrane library with language restrictions to English for original research articles, systematic reviews and meta-analyses to identify all available literature on vernakalant and atrial fibrillation. We combined search terms for atrial fibrillation with vernakalant or RSD1235.

Inclusion criteria required each study to be randomized controlled trial comparing intravenous infusion of vernakalant with placebo or an alternative antiarrhythmic drug to treat AF including recent onset of AF, paroxysmal AF. We excluded non-randomized studies, those involving children or animals and also trials that evaluated the prophylactic use of vernakalant to prevent rather than to treat AF. This meta-analysis followed through The Preferred Reporting Items for Systematic Reviews and Meta-Analyses: The PRISMA Statement [9].

### Data extraction

One author performed the literature search, and data extraction was independently conducted by two individuals. The following information was extracted: publication details, timing of study, duration of follow-up, randomization method, blinding (of participants, investigators, and outcome assessors), vernakalant dosage and route of administration, dropouts, mean age of participants, primary outcome (conversion rate to sinus rhythm) and secondary outcome (adverse events of cardiac origin). Any disagreements in the collected data was elucidated by consensus or, if necessary, upon consultation with a third reviewer. The authors of the original publications were contacted to obtain missing data.

Quality assessment of the individual trials was performed using Jadad scale [10] (range from 0 to 5, with the higher scales indicating a better quality trial). The grading of allocation concealment was according to the Cochrane approach, that is, adequate or uncertain or clearly inadequate. Data were checked and entered into the Review Manager 5 for Windows.

### Outcomes of interest

The proportion of patients with atrial fibrillation converted to sinus rhythm within different duration of treatment was chosen as the primary outcome because it is the most relevant clinical outcome in patients with AF. And the occurrences of adverse events between vernakalant and the control groups were also assessed. There was no missing data for the main outcomes in the trials included.

### Statistical analysis

The differences in categorical outcomes between vernakalant and placebo or an alternative antiarrhythmic drug were reported as risk ratio (RR) with 95% confidence interval (CI), using a fixed effect model. The differences in adverse effects of cardiac origin between vernakalant and control groups were assessed by RR with 95% CI using a random effect model. The presence of heterogeneity between trials was assessed by the X^2^ statistics and the extent of inconsistency was assessed using I^2^ statistics. All tests were two-tailed with a P value of less than 0.05 as the level of statistical significance.

## Results

### Study selection and description

**Figure 1:**
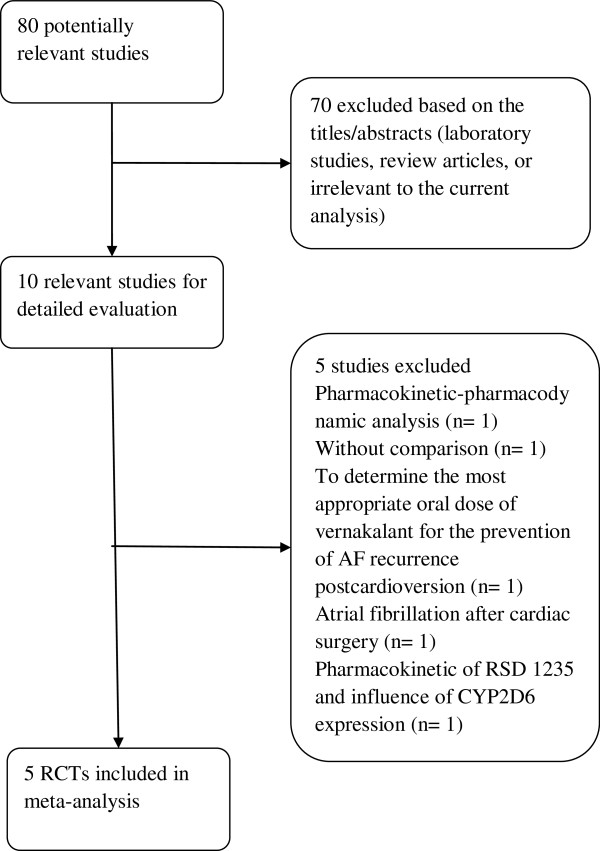
Flow diagram of selection process of randomized controlled trials included in meta-analysis.

Our electronic searches identified 80 studies of which 5 articles [7,8,11-13] fulfilled our inclusion criteria and were subjected to meta-analysis (Figure 1). The 5 included trials were published in english. There was complete agreement on inclusion assessment between the three reviewers. Table 1 showed the characteristics of the 5 included trials. Four trials [7,8,12,13] compared vernakalant with placebo and the remaining one [11] was with amiodarone.

**Table 1:** General characteristics of included studies

**Study**	**N**	**Treatment groups**				**Subject characteristics**		**No. with conversion**	
		**Regimen**	**n**	**Goal follow-up time**	**Atrial fibrillation time**	**Age (Years)**	**Men (%)**	**n/N**	**%**
Camm 2011	232	Vernakalant	116	1 week	AF 3 to 48	63	63	60/116	51.7%
		3 mg/kg IVI f/b			hrs				
		2 mg/kg if							
		required 15 min							
		later						6/116	5.2%
		Amiodarone 5 mg/kg 60-min IVI f/b 50 mg IVI over additional 60 min	116						
Pratt 2010	239	Vernakalant	118	30 days	AF 3hrs to	63	65	47/118	40%
		3 mg/kg IVI f/b			45 days				
		2 mg/kg if							
		required 15 min						4/121	3%
		later							
		Placebo	121						
Roy 2004	56	Vernakalant	18	1 week	AF 3 to 72	64	61	2/18	11%
		0.5 mg/kg IVI f/b			hrs				
		1.0 mg/kg if							
		required 30min	18					11/18	61%
		later							
		Vernakalant							
		2.0 mg/kg IVI f/b 3.0 mg/kg if required 30 min later Placebo	20					1/20	5%
Roy 2008	336	Vernakalant	221	30 days	AF 3 hrs	62	69	83/221	37.6%
		3 mg/kg IVI f/b			to 45 days				
		2 mg/kg if							
		required 15 min							
		later						3/115	2.6%
		Placebo	115						
Stiell 2010	290	Vernakalant	229		AF 3 to 48		69	136/229	59.4%
		3 mg/kg IVI f/b			hrs				
		2 mg/kg if		1 week		59			
		required 15 min							
		later	61					3/61	4.9%
		Placebo							

**Table 2:** Adverse effects

**Study name**	**T/C**	**Adverse events**	
		**Type**	**T/C**
Camm 2011 [11]	116/116	Monomorphic nonsustained	1/0
		ventricular tachycardia	
		Cardiac arrest	0/1
Pratt 2010 [7]	118/121	Ventricular tachycardia	16/13
		Ventricular fibrillation	1/0
		Bradycardia	8/0
		Hypotension	7/2
Roy 2004 [12]	36/20	Cardiac disorders	7/7
Roy 2008 [8]	221/115	Nonsustained ventricular tachycardia	14/17
		Bradycardia	1/0
		Hypotension	14/4
		Ventricular bigeminy	1/0
		Uncorrected QT > 550 ms	1/1
Stiell 2010 [13]	229/61	Any ventricular arrhythmia events	20/10
		Ventricular tachycardia	18/9
		Ventricular extrasystoles	2/1
		Bradycardia	21/7
		Hypotension	20/7

The doses of intravenous vernakalant used ranged from 0.5 mg/kg to 3 mg/kg. The time period assessed for the selected clinical end point of vernakalant treatment ranged between 30-min after last infusion to 90-min of drug initiation.

### Assessment of validity

The quality of the included trials varied; the Jadad scale [10] ranged from 1 to 4 (mean 3). All trials were randomized but there was one trial which was not double-blinded. The proportion of patients lost or excluded was less than 10% in all the included trials. The agreement between reviewers was over 90% for different criteria.

### Effect of intravenous vernakalant on rhythm conversion

**Figure 2:**
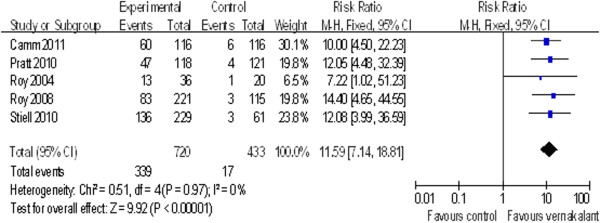
Forest Plot of the Risk Ratio of vernakalant on conversion rate of AF to SR.

Five trials involving 1153 patients reported data on the effect of intravenous vernakalant on rhythm conversion. The relative risk (RR) of vernakalant on conversion rate of AF to SR was 11.56 (95% confidence interval [CI] 7.12 to 18.75; p<0.00001) (Figure 2). Figure 2 depicts the RRs and 95% CI for conversion to SR with vernakalant compared with control treatments for each of the 5 studies and 1 pooled RR.

### Adverse effects of cardiac origin

**Figure 3:**
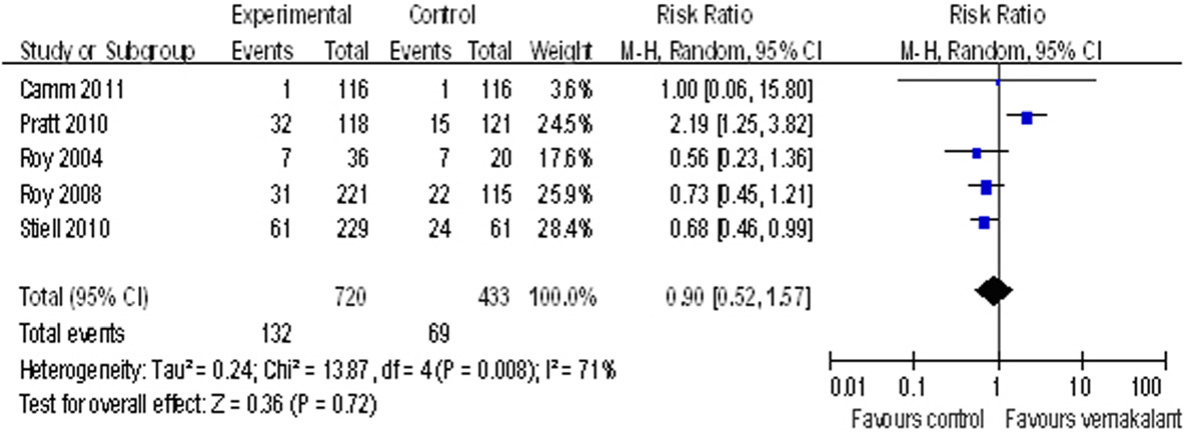
Forest Plot of the Risk Ratio of vernakalant on adverse events.

All five trials included in this study showed adverse events (AEs) of cardiac origin. There was heterogeneity among the AEs of these studies (I^2^=71%), and thus with random effect model, the RR of vernakalant on AEs of cardiac origin was 0.90 (95%CI=0.52 to 1.57; p=0.72) (Figure 3).

#### Sensitivity analyses

A series of sensitivity analyses was done to confirm the same directionality for the efficacy end point (conversion to SR) and the safety end point (adverse events of cardiac origin). There was no individual study affected the overall results for both efficacy and safety end points.

## Discussion

This meta-analysis assessed the efficacy and safety of vernakalant on conversion of AF to SR. Vernakalant is associated with a stastically significant success in conversion of AF to SR. In this meta-analysis, vernakalant was superior to placebo and amiodarone in converting AF to SR, but there was no difference in adverse effects of cardiac origin between the two groups.

There was one trial [13] with low quality had included into our analysis, obtaining 1 point in the Jadad scale. This study was not excluded because that paper was a post hoc analysis of ACT I and ACT IV in which ACT I was double-blind while the later was open-label. The remaining 4 trials had Jadad scale of 3 [7] and 4 [8,11,12] respectively. The meta-analysis of vernakalant efficacy as compared with placebo or amiodarone revealed no heterogeneity (I^2^=0%) while there was significant heterogeneity in comparison of AEs of cardiac origin between the two groups (I^2^=71%). This may be mainly due to low incidence of AEs of cardiac origin in one trial [11] and the differences of follow-up time ranging from 1 week to 30 days.

The result of this meta-analysis is in accordance with previous reviews [14,15] showing vernakalant is superior to placebo and amiodarone. It is needed to state that the conversion rates with vernakalant were lower in the AVRO [11] trial than those in a post hoc analysis [13] of data from ACT I and ACT IV in 3–48 hours’ duration AF patients (51.7% vs 59%) which may be caused by the different medical history, having a higher propotion of patients with CHF included in the AVRO trial (20%) than later study (5%) [11,13]. Vernakalant is also superior in achieving SR in patients with post-operative AF enrolled in ACT II as compared with placebo (47% vs 14%) [16]. The highest efficacy of vernakalant cardioversion rate was observed for AF up to 72 hours (70–80%) [14] while it was relatively ineffective in AF patients with duration more than 7 days and in atrial flutter, having conversion rates of 8% and 2.5% respectively in ACT I and 9 and 7% respectively in ACT III. Promising results were also obtained by evaluating an oral formulation of vernakalant for the maintenance of SR in phase II, placeo-controlled, dose-ranging studies (300 mg or 600 mg twice daily) [17]. Vernakalant could achieve rapid termination of AF with median conversion time ranging from 8 min to 12 min while the median conversion time in control groups was much longer over 30 min to over 100 min.

This meta-analysis showed no significant difference in occurring of AEs of cardiac origin between the two arms. The AEs other than cardiac origin was not extracted and analysed due to the presence of patients reporting more than one adverse event. According the trials, the most common drug-related adverse events occurring within the first 24 hours were dysgeusia, sneezing, paraesthesia and cough. Common cardiac disorders resulted in vernakalant groups included bradycardia and atrial flutter. The previous one responded well to discontinuation of vernakalant and/or intravenous atropine while the later was converted to SR with continuation of the infusion of vernakalant. Vernakalant is generally well tolerated in conversion of AF to SR with no significant QTc prolongation and proarrhythmia with the use of this drug have not been reported to date.

### Limitations of this study

This study has some limitations. As our paper aimed to include only English full-text articles published in peer-reviewed journals, there is a potential for publication bias. The number of included studies was too low to perform publication bias assessment using funnel plot or other widely accepted statistical methods. However, we can expect that studies excluded from the analysis were small and of low quality. As low-quality studies comprised less than 2% of the whole patient population included into the meta‑analysis, the potential effect of lacking studies should be very low. Our study showed that vernakalant is superior to control group and since there was only one study comparing with amiodarone was included in this meta-analysis, further research comparing vernakalant and other antiarrhythmic agents through direct head-to-head comparisons may be needed to confirm this effect. There was no difference shown in safety outcome in this study between the vernakalant and control groups, however, more randomized controlled trials with a longer follow-up are needed to better evaluate the efficacy and safety of vernakalant on atrial fibrillation patients.

## Conclusions

Vernakalant is effective and relatively rapid acting in converting AF to SR in a wide range of patients. Intravenous Vernakalant when compared to other antiarrhythmic agents is effective in converting AF to SR and may be used as first-line therapy in patients with AF.

## Competing interests

The authors declare that they have no competing interests.

## Authors’ contributions

All authors were equally contributed. All authors read and approved the final manuscript.
